# Absorption, accumulation and metabolism of cetoleic acid from dietary herring oil in tissues of male Zucker Diabetic Sprague Dawley rats

**DOI:** 10.1017/S0007114525000236

**Published:** 2025-03-14

**Authors:** Eira V. Rimmen, Svein Are Mjøs, Eirik Søfteland, Oddrun A. Gudbrandsen

**Affiliations:** 1 Dietary Protein Research Group, Centre for Nutrition, Department of Clinical Medicine, University of Bergen, Bergen 5021, Norway; 2 Department of Chemistry, University of Bergen, Bergen 5020, Norway; 3 Department of Medicine, Haukeland University Hospital, Bergen, Norway

**Keywords:** Cetoleic acid, *n*-11 MUFA, EPA, DHA, Desaturases

## Abstract

Accumulation of exogenous fatty acids such as the long-chain *n*-11 MUFA cetoleic acid (CA, C22:1*n*-11) may induce functional changes, through direct effects or by affecting the amounts of other fatty acids through changes in catabolic and anabolic processes including desaturation of fatty acids or by other processes. The primary aim of this study was to investigate if dietary CA was absorbed and accumulated in a TAG-rich tissue for storage (white adipose tissue), a stable phospholipid-rich tissue (brain), metabolically active tissues (liver and skeletal muscle) or circulating in the blood (blood cells) and metabolised. Secondary aims included investigating any effects on the levels of EPA and DHA. Eighteen male Zucker diabetic Sprague Dawley (ZDSD) rats were fed diets with herring oil (HERO) containing 0·70 % CA or anchovy oil (ANCO) devoid of CA, or a control diet with soyabean oil for 5 weeks. The HERO and ANCO diets contained 0·35 and 0·37 wt% EPA + DHA, respectively. Data were analysed using one-way ANOVA. CA from dietary HERO was absorbed, and CA and two chain-shortened metabolites were found in blood cells, liver, white adipose tissue (WAT) and muscle, but *n*-11 MUFAs were not found in the brain. The concentrations of EPA and DHA were similar in liver lipids (TAG, cholesteryl esters and NEFA) as well as in WAT, muscle and brain from rats fed the HERO or ANCO diets. To conclude, CA was taken up by tissues but did not affect levels of EPA and DHA in this diabetic rat model.

Vertebrates have limited capacity to biosynthesise the long-chain MUFA cetoleic acid (CA, C22:1*n*-11); however, CA is found in high levels in fish species feeding on zooplankton such as copepods^(1)^. Unlike the well-known and much-studied long-chain *n*-3 PUFAs EPA and DHA that are also found in fish and other marine species, few studies have investigated the metabolism of CA or the metabolic effects of CA intake in vertebrates. Research on the metabolism of ingested CA in humans and other animals is limited, but studies in grey seals (*Halichoerus grypus*) and minks (*Mustela vison*) demonstrate that CA is chain-shortened in peroxisomes by oxidation to C20:1*n*-11, C18:1*n*-11 and C16:1*n*-11 and is further oxidised in mitochondria to acetyl groups to be used for *de novo* fatty acid synthesis^(2)^. Findings in cell culture studies suggest that a beneficial effect of CA may be through stimulation of the endogenous synthesis of EPA and DHA from *α*-linolenic acid by upregulating fatty acid desaturases 1 and 2 (FADS1 and FADS2) in the liver^(3)^. EPA and DHA that are incorporated in phospholipids (PL) in membranes may affect the fluidity and thus the function of the cells, and they are precursors for anti-inflammatory prostaglandins, leukotrienes, resolvins and protektins^(4)^. Others have suggested that CA has a beneficial effect on the metabolism by stimulating the oxidation of other fatty acids, based on the observation of a higher oxidation *ex vivo* in peroxisomes^(5)^, but not in mitochondria^(6)^, from liver cells isolated from Wistar rats fed a diet with a high content of long-chain MUFA.

We recently summarised and meta-analysed the available literature investigating the effects of the consumption of fish oils or fish oil concentrates with a high content of CA but low or no content of EPA and DHA on the circulating cholesterol concentration in rodents, showing that CA-rich fish oils and concentrates prevent high cholesterol concentration^(7)^. The articles included in the systematic review did not provide any details on the mechanism(s) behind the lower cholesterol concentration after CA intake^(7)^. A recently published paper from our group showed that when Zucker diabetic Sprague Dawley (ZDSD) rats were fed a diet containing herring oil, which contains a high amount of CA but has relatively low contents of both EPA and DHA compared with anchovy oil (ANCO), this resulted in a lower serum cholesterol concentration compared with a control group fed a diet containing soya oil^(8)^, indicating that CA and possibly its metabolites were taken up by the tissues to induce metabolic effects. This is an interesting finding since elevated circulating cholesterol concentration is a major risk factor for CVD^(9,10)^, the leading cause of disease burden globally^(11)^. The lower serum cholesterol concentration was accompanied by a higher faecal excretion of bile acids, whereas markers of the cholesterol production in the liver, the hepatic secretion of VLDL and the liver’s capacity to take up cholesterol were similar to controls^(8)^.

The knowledge on the metabolism of CA is scarce, and more information on where CA is stored in the body is warranted to better understand any impact of CA consumption on biochemical and physiological processes, including the reported lowering of cholesterol^(7,8)^. Therefore, the main aim of this study was to investigate if CA and any elongated or chain-shortened metabolites of CA are accumulated in a TAG-rich tissue for storage (WAT), a stable PL-rich tissue (brain), metabolically active tissues such as the liver and skeletal muscle or circulating in the blood (blood cells) in ZDSD rats fed a diet containing fish oil with CA. Our hypothesis was that CA would be recovered in all examined tissues. The secondary aims were to investigate any effects on the levels of other fatty acids including EPA and DHA in these tissues and liver lipids and to explore any differences in the levels of the desaturases FADS1 and FADS2 in liver and skeletal muscle in ZDSD rats consuming diets containing fish oils with or without CA. To achieve this, the fatty acid compositions in adipose tissue (mainly TAG for storage), the brain (mainly PL and cholesterol, quite stable composition), skeletal muscle (relatively high in PL but also contains other lipids) and blood cells (mainly PL, less stable) were mapped in rats consuming diets added herring oil or anchovy oil, containing 0·35 and 0·37 wt% EPA + DHA, respectively. In addition, PL, TAG, cholesteryl esters (CE) and NEFA in the liver were separated before the fatty acids were quantified, and the levels of desaturases in the liver and skeletal muscle were measured. Since herring oil contains *n*-3 long-chain-PUFAs, we used anchovy oil with a similar EPA + DHA content to control for dietary intake of EPA and DHA and the endogenous synthesis of the long-chain *n*-3 PUFAs.

## Methods

### Animals and diets

The design of this study including housing conditions and the diets has been described in detail in Rimmen et al.^(8)^, and the present study presents secondary analyses of this experiment. Twenty-four male (ZDSD/PcoCrl) rats were obtained from Charles River. The ZDSD rat model was generated by crossing the Zucker Diabetic Fatty (Lean +/+) with Sprague–Dawley Crl:CD rats^(12)^. The ZDSD rat has an intact leptin signalling pathway and develops polygenetic metabolic disturbances with insulin resistance resulting in type 2 diabetes that progresses similarly to the disease in humans including the destruction of pancreatic *β*-islet cells^(12,13)^. The rats were 81–95 d old at arrival and were housed in pairs in 1500U Eurostandard Type IV S cages (IVC Blue Line, Tecniplast) with temperature 22–23°C, in a room with controlled light/dark cycle (dark 20.00–06.00). The rats were fed standard chow as used in our animal facility (V1536, containing 19·1 % protein, 3·6 % fat, 4·8 % sugar, from ssniff Spezialdiäten GmbH, Soest, Germany) until they were approximately 16 weeks of age. Rats were fed a higher fat diet to synchronise the onset of type 2 diabetes, as recommended by Charles River^(14)^, using an in-house diet containing 20wt% protein (casein), 10wt% sucrose, 16·2wt% maltose dextrin, 32wt% lard and 7wt% soyabean oil. See Rimmen et al.^(8)^ for more details.

Eighteen of the rats (75 %) developed diabetes (blood glucose > 13·9 mmol/l) and were randomly assigned to receive one of the three experimental diets by drawing paper lots from a jar. Each group consisted of six rats, and the rats were given random numbers that could not be linked to the experimental group. The rat cages were randomly placed in the rack. The experimental semi-purified diets were modified versions of the American Institute of Nutrition’s recommendation for growing laboratory rodents (AIN-93G)^(15)^ with the addition of 1·6 g methionine/kg diet as recommended by Reeves^(16)^ and differed only in their lipid sources (Table 1). All diets contained 20wt% protein (casein), 10wt% fat and 11·8wt% sucrose and had comparable energy content^(8)^. We chose to use the AIN-93G diet instead of the AIN-93M diet for maintenance containing 15wt% protein since the ZDSD rats will develop insulin resistance. Insulin resistance leads to increased muscle protein breakdown in both rodents and humans^(17,18)^. The two intervention diets contained either refined oil prepared from herring (*Clupea harengus*) residuals (the HERO diet) or refined oil prepared from whole anchovies (*Engraulidae*) (the ANCO diet), and were designed with a comparable content of EPA + DHA but different CA content. All diets contained adequate amounts of essential fatty acids according to Reeves et al.^(15)^. The control diet contained soyabean oil as the only lipid source. All ingredients were purchased from Dyets Inc. (Bethlehem, PA, USA) except casein which was purchased from Sigma-Aldrich (Munich, Germany), herring oil from Pelagia AS (Bergen, Norway) and anchovy oil from Epax Norway AS (Ålesund, Norway). The rats had ad libitum access to feed and water in their home cage. The diets were stored at –15°C, and daily portions were thawed in the morning.

**Table 1.** Compositions of the experimental diets

Contents (g/100g diet)	Control diet	HERO diet	ANCO diet
Soyabean oil	10·00	6·30	8·40
Herring oil	–	3·70	–
Anchovy oil	–	–	1·60
Casein^*^	21·56	21·56	21·56
Sucrose	9·00	9·00	9·00
Cornstarch	48·23	48·23	48·23
Cellulose	5·00	5·00	5·00
t-Butylhydroquinone	0·0014	0·0014	0·0014
Mineral Mix (AIN-93-MX)^**^	3·50	3·50	3·50
Vitamin Mix (AIN-93-VX)^†^	1·00	1·00	1·00
Growth and maintenance supplement^‡^	1·00	1·00	1·00
L-Methionine	0·16	0·16	0·16
L-Cystine	0·30	0·30	0·30
Choline Bitartrate^§^	0·25	0·25	0·25

HERO, herring oil; ANCO, anchovy oil.

* Contains 91·9 % crude protein (high-fat diet) or 92·78 % crude protein (other diets).

** Contains sucrose (221 g/kg).

† Contains sucrose (967 g/kg).

‡ Contains vitamin B12 (40 mg/kg) and vitamin K1 (25 mg/kg) mixed with sucrose (995 g/kg) and dextrose (5 g/kg).

§ Contains 41 % choline.

The rats were housed in IVC equipped with one gnawing block (Aspen brick, size 100 mm × 20 mm × 20 mm, TAPVEI® Harjumaa, Estonia OÜ), three paper sachets containing softwood bedding for nesting material (2HK Nestpak, Datesand Ltd, Manchester, UK) and one red polycarbonate hut (Fat Rat Hut, size 150 mm × 165 mm × 85 mm, Datesand). Due to signs of behavioural changes during the second week of housing in IVC, rats had periodic stays in a large ‘playcage’ with the purpose to increase their well-being and provide cognitive as well as physical stimulation, as described in detail elsewhere^(19)^.

### Design

Rats were weighed three times per week, The groups had similar body weight at baseline (mean 542 (sd 36) grams, *P* ANOVA 0·80), and the reduction in body weight during the intervention period was similar between the groups (mean −66 (sd 22) grams, *P* ANOVA 0·74), as previously presented^(8)^. At the end of the experimental period, that is, after 36–37 d with powder feed, the feed was withdrawn at 06.30. The rats were fasted for 6 h, with free access to drinking water, and were killed while anaesthetised with isoflurane (Isoba vet, Intervet, Schering-Plough Animal Health, Boxmeer, The Netherlands) mixed with oxygen. Blood was drawn from the heart and collected in BD Vacutainer K2EDTA tubes (Becton, Dickinson and Company) for separation of blood cells from plasma. The blood cell fraction was frozen at −80°C. Liver, epididymal white adipose tissues (WATepi), skeletal muscle from the thigh and brain were carefully dissected and frozen at −80°C.

The personnel handling and conducting the analyses were blinded to the rats’ group allocation. The rats were handled and killed in random order.

### Determination of fatty acid compositions in diets and tissues

Lipids were extracted from diets, liver and thigh muscle by the method of Bligh and Dyer^(20)^ using a mixture of chloroform and methanol, and extracts were added to heneicosanoic acid (C21:0) as internal standard and were methylated, as described previously^(21)^. Lipid classes in liver and fish oils were separated by TLC on silica gel plates (250 um Silica gel 60 from Merck KGaA, Darmstadt, Germany) developed in hexane–diethyl ether–acetic acid (40:10:1, by vol)^(22)^. The liver TAG, PL, CE and NEFA spots were identified using Rhodamine G (Fluka Chemie AG, Buchs, Switzerland) and co-migration with known standards, and were scraped off, added to heneicosanoic acid (C21:0) as internal standard and were methylated, as described previously^(21)^. Samples of blood cells, WATepi and brain were added to heneicosanoic acid (C21:0) as internal standard and were methylated without prior extraction of lipids, as previously described^(21)^. The methyl esters in the samples were quantified in randomised order using an Agilent 7890 gas chromatograph equipped with a flame ionisation detector (Agilent Technologies, Inc.) and a BPX-70 capillary column (SGE Analytical Science) as described in Sciotto & Mjøs^(23)^ with minor adjustments of the temperature programme to provide baseline resolution between *n*-9 and *n*-11 MUFA isomers. To assure accurate quantitative amounts, chromatographic areas were adjusted with empirical response factors based on the GLC-793 reference mixture (Nu-Chek Prep, Elysian, MN, USA). The reference mixture was run as every eighth sample (or more often) in the chromatographic sequences, and each sequence included at least four samples of the reference mixture. The fatty acids in the reference mixture were identified by GC-MS using the methodology described in Wasta & Mjøs^(24)^.

### Protein analyses in liver and skeletal muscle

Liver and thigh muscle samples were homogenised in PBS, and protein was quantified with the Bradford dye-binding method^(25)^ using protein assay dye reagent (Bio-Rad Laboratories, Munich, Germany) with bovine serum albumin (Bio-Rad Protein Assay Standard II, Bio-Rad Laboratories, Hercules, CA, USA) as the standard. Fatty acid desaturase 2 (FADS2, also known as delta-6 desaturase) was measured using the Rat Delta-6 Desaturase/FADS2 ELISA Kit (Sandwich ELISA) cat no LS-F7004 (LifeSpan BioSciences, Inc.). Fatty acid desaturase 1 (FADS1, also known as delta-5 desaturase) was measured using the Rat FADS1 ELISA Kit (Sandwich ELISA) cat no LS-F56186 (LifeSpan BioSciences, Inc.). Stearoyl-CoA desaturase 1 (SCD1, also known as delta-9 desaturase) was measured using the Rat SCD1/SCD ELISA Kit (Sandwich ELISA) cat no LS-F32611 (LifeSpan BioSciences, Inc.). Plates were read at 450 nm on a SpectraMax Plus384 Microplate Reader. All samples were analysed simultaneously in the same plate from each of the ELISA assays, with CV < 5 %, Concentrations of FADS2, FADS1 and SCD1 in the liver and concentrations of FADS2 and FADS1 in muscle are presented relative to protein content.

### Outcome measurements

The primary outcome was to investigate in which organs CA and its metabolites can be found after consuming a diet containing fish oil with CA. The secondary outcomes were to investigate any effects on the levels of other fatty acids including EPA and DHA and to explore any differences in the levels of the desaturases FADS1 and FADS2 in liver and skeletal muscle.

### Statistical analyses

This study was primarily designed to investigate the effects of fish oil consumption in ZDSD rats^(8)^. Since this was the first study designed with this outcome in ZDSD rats, no data on effect size were available for sample size calculation or minimally detectable effect sizes. Based on studies conducted in rats and mice using CA-rich fish oils with group sizes of 6–12 rodents/group^(7)^, the study was designed with eight rats per experimental group. We expected all rats to become diabetic when fed the high-fat diet; however, 25 % of the rats did not develop diabetes. Therefore, statistical analyses are conducted with *n* 6 rats in each of the experimental groups.

Statistical analyses were conducted using SPSS Statistics version 28 (SPSS, Inc., IBM Company). All data were evaluated for normality using the Shapiro–Wilks test, revealing that the majority variables, with the exception of a few fatty acids that were found in very low amounts, were normally distributed; therefore, one-way ANOVA was used to compare the experimental groups. Datasets with non-parametric distribution were log-transformed before analyses. Since this study is regarded as an exploratory study without the possibility of a proper calculation of the necessary sample size, when appropriate, the ANOVA analyses were followed by Tukey HSD post hoc test as recommended by Lee et al.^(26)^. The cut-off value for statistical significance was set at a probability of 0·05.

## Results

### Fatty acids in the diets

CA and gadoleic acid (GA, C20:1*n*-11) were found solely in the HERO diet, whereas a small amount of 7-octadecenoic acid (7OA, C18:1*n*-11) was found in both the HERO diet and the ANCO diet (Table 2). The *n*-3 PUFA stearidonic acid (C18:4 *n*-3), EPA, *n*-3 docosapentaenoic acid (DPA, C22:5*n*-3) and DHA were found in the HERO diet and in the ANCO diet, with comparable amount of EPA + DHA. Long-chain *n*-11 MUFAs and long-chain *n*-3 PUFAs were not found in the Control diet. The essential fatty acids linoleic acid (C18:2*n*-6) and *α*-linolenic acid (C18:3*n*-3), as well as oleic acid (C18:1*n*-9), all of which mainly originate from the soya oil added to the diets, were found in all three diets. The contents of saturated fatty acids were in general similar between the diets, i.e. deviation less than 0·1g/100 g diet, with the exception of myristic acid (C14:0) which was lowest in the Control diet and highest in the HERO diet. The long-chain MUFA gondoic acid (C20:1*n*-9) was found in the highest amount in the HERO diet compared with the other diets, and the longer chain *n*-9 MUFA erucic acid (C22:1*n*-9) and nervonic acid (C24:1*n*-9) were identified only in the HERO diet. The fish oils consisted mainly of TAG, with only 0·25 and 0·04 wt% of fatty acids esterified as PL in the herring oil and the anchovy oil, respectively.

**Table 2.** Contents of fatty acids in the diets

g/100g diet	Control diet	HERO diet	ANCO diet
C14:0	0·02	0·26	0·12
C15:0	<LOQ	0·02	0·01
C16:0	0·92	0·95	0·98
C17:0	0·01	0·01	0·01
C18:0	0·31	0·24	0·30
C20:0	0·02	0·02	0·02
C22:0	0·02	0·02	0·02
C16:1 *n*-7	0·01	0·13	0·11
C18:1 *n*-5	<LOQ	0·01	0·01
C18:1 *n*-7	0·12	0·11	0·13
C18:1 *n*-9	1·71	1·31	1·51
C20:1 *n*-9	0·01	0·35	0·02
C22:1 *n*-9	<LOQ	0·03	<LOQ
C24:1 *n*-9	<LOQ	0·03	<LOQ
C18:1 *n*-11	<LOQ	0·04	0·03
C20:1 *n*-11	<LOQ	0·05	<LOQ
C22:1 *n*-11	<LOQ	0·70	<LOQ
C16:2 *n*-4	<LOQ	0·02	0·02
C16:3 *n*-4	<LOQ	0·01	0·02
C18:2 *n*-6	4·52	2·92	3·73
C18:3 *n*-3	0·57	0·39	0·48
C18:4 *n*-3	<LOQ	0·07	0·04
C20:5 *n*-3	<LOQ	0·17	0·23
C22:5 *n*-3	<LOQ	0·02	0·02
C22:6 *n*-3	<LOQ	0·18	0·14

HERO, herring oil; ANCO, anchovy oil; LOQ: level of quantification.

The following fatty acids were below LOQ in all diets: C12:0, C23:0, C24:0, C14:1 *n*-5, C16:1 *n*-9, C17:1*n*-7, C17:1*n*-8, C18:1*n*-9t, C20:1*n*-7, C22:1*n*-7, C18:2*n*-6tc, C18:3*n*-6, C18:2 *n*-4, C20:2*n*-6, C20:3*n*-6, C20:4*n*-6, C20:3*n*-3, C20:4 *n*-3, C21:5 *n*-3, C22:4*n*-6 and C22:5*n*-6.

### CA and its metabolites

**Figure 1.**
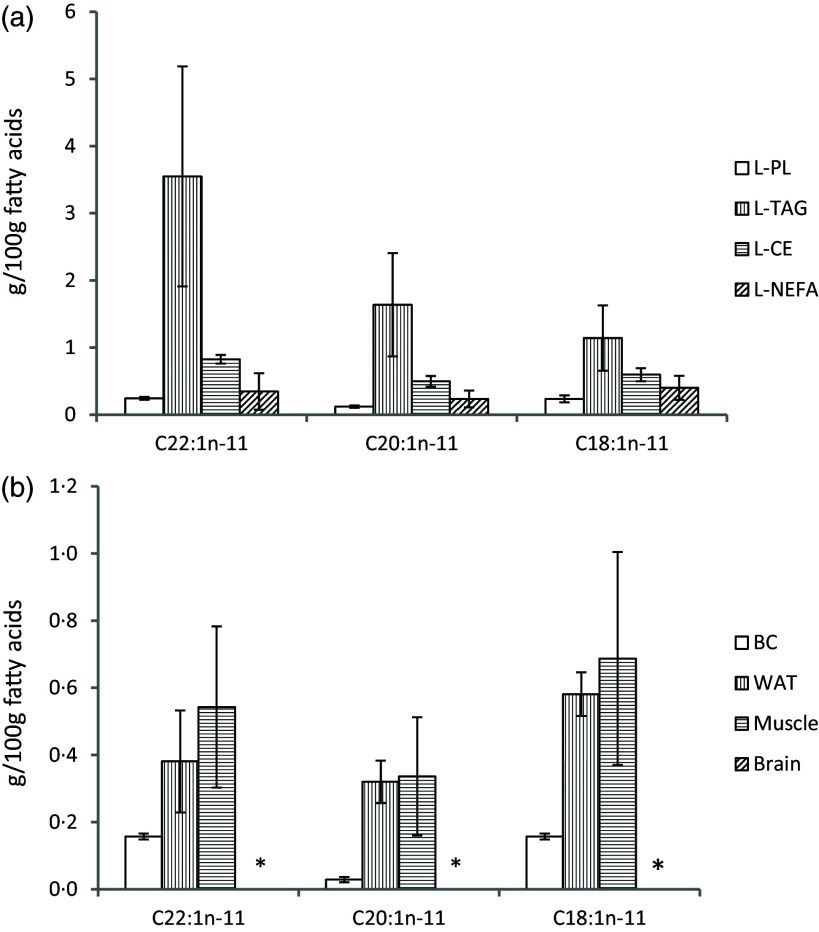
Relative weights of *n*-11 MUFAs in liver lipids (g/100g FAs) for phospholipids (PL), TAG, cholesteryl esters (CE) and as NEFA (a) and in total lipids from blood cells (BC), epididymal white adipose tissue (WAT), skeletal muscle (muscle) and brain (b) from rats fed the HERO diet. *n*-11 MUFAs were not recovered in rats fed the ANCO diet or the control diet. Data are presented as mean and standard deviation for *n* 6 rats in each experimental group. HERO, herring oil; ANCO, anchovy oil. * CA, GA and 7OH were not detected in the brain.

Liver lipids were separated into PL, TAG, CE and NEFA by using TLC. The *n*-11 MUFAs CA, GA and 7OA were identified in all lipid classes from rats fed the HERO diet, with the highest relative amounts of all three *n*-11 MUFA found in liver TAG (Figure 1(a), online Supplementary Tables 1–4). We did not detect any *n*-11 long-chain MUFAs with chain length > 22 carbons. CA, GA and 7OA were not detected in liver lipids from rats in the control group or in the ANCO group.

CA, GA and 7OA were recovered in blood cells, epididymal white adipose tissue and skeletal muscle, but not in the brain, from rats fed the HERO diet (Figure 1(b), online Supplementary Tables 5–8). In control rats and rats in the ANCO group, CA, GA and 7OA were not detected in blood cells, WATepi, skeletal muscle or the brain. The relative amount of CA in extrahepatic tissues ranged from zero in the brain, to mean 0·543 (sd 0·240) g/100 FA in muscle.

### Long-Chain *n*-3 PUFA

**Figure 2.**
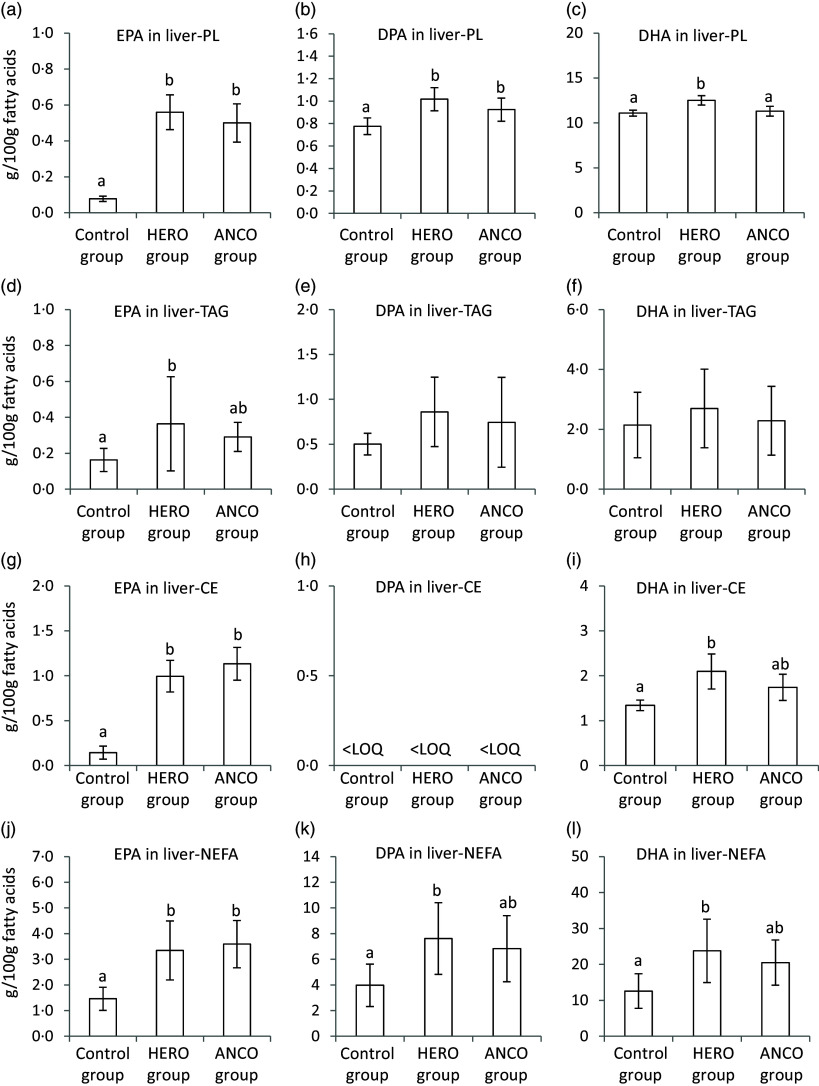
Relative weights (g/100g FAs) of EPA, DPA and DHA in liver lipids for phospholipids (L-PL) (a–c), L-TAG (d–f), cholesteryl esters (L-CE) (g–i) and as L-NEFA (j–l). Data are presented as mean and standard deviation for *n* 6 rats in each experimental group. Groups are compared using one-way ANOVA followed by Tukey HSD post hoc test when appropriate. Bars with different letters are significantly different (*P* < 0·05). HERO, herring oil; ANCO, anchovy oil; LOQ, level of quantification.

The relative amount (g/100 g FA) of EPA was higher in PL, TAG, CE and NEFA from the liver of rats fed the HERO diet when compared with the control group (Figure 2, online Supplementary Tables 1–4). The relative amount of DHA was higher in liver PL, liver CE and liver NEFA in the HERO group, whereas the DHA concentration in liver TAG was similar to that of the control group. The DPA amount was higher in liver PL and liver NEFA in rats fed the HERO diet and similar in liver TAG to the control group but DPA was not detected in liver CE. The contents of EPA, DHA and DPA were similar within the respective liver lipids between the HERO group and the ANCO group, with the exception of a higher DHA content in liver PL in the HERO group compared with the ANCO group. The absolute amounts of EPA, DPA and DHA in the liver were calculated by summarising the amounts of these fatty acids esterified as PL, TAG or CE, or as NEFA (online Supplementary Figure). The total amounts of EPA and DPA in the liver were higher in the HERO and ANCO groups compared with the control group, with no difference between the HERO and ANCO groups. The total DHA amount in the liver was higher in the HERO group compared with both control and ANCO groups, with no difference between the two latter groups.

**Figure 3.**
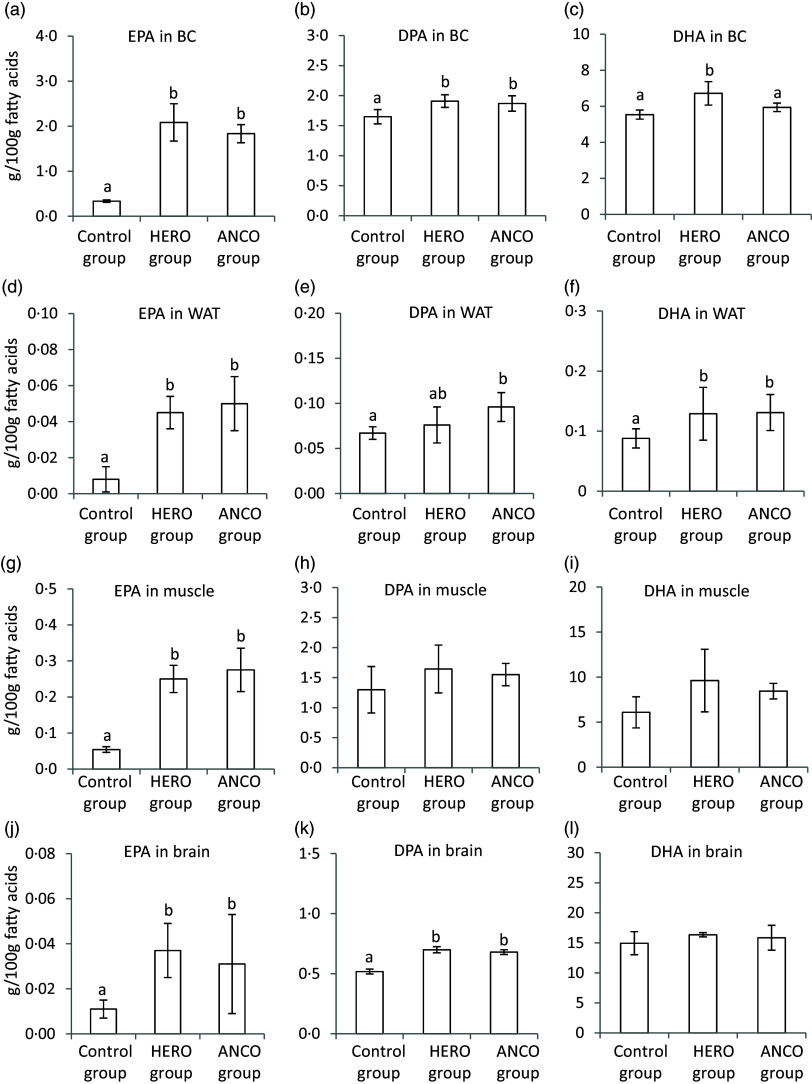
Relative weights (g/100g FAs) of EPA, DPA and DHA in total lipids from blood cells (BC) (a–c), epididymal white adipose tissue (WAT) (d–f), skeletal muscle (muscle) (g–i) and brain (j–l). Data are presented as mean and standard deviation for *n* 6 rats in each experimental group. Groups are compared using one-way ANOVA followed by Tukey HSD post hoc test when appropriate. Bars with different letters are significantly different (*P* < 0·05). HERO, herring oil; ANCO, anchovy oil.

The content of EPA was significantly higher in blood cells, WATepi, muscle and brain from rats fed the HERO diet or the ANCO diet, with no difference between the two groups, when compared with the Control group (Figure 3, online Supplementary Tables 5–8). The DPA content was higher in blood cells and brain in both HERO and ANCO groups when compared with controls, whereas the level of DPA in muscle was similar for all three groups and significantly higher in WAT harvested from the ANCO group. The DHA content was higher in blood cells from the HERO group and in WAT from both HERO and ANCO groups but was not affected by the fish oil diets in muscle and brain. The levels of EPA, DPA and DHA were similar between the HERO and ANCO groups for WAT, muscle and brain. In blood cells, the HERO and ANCO groups were similar with regard to the amounts of EPA and DPA, whereas the DHA amount was higher in the HERO group when compared with both the ANCO group and the control group.

### Other MUFAs of interest

Oils from fish that feed on copepods are rich in gondoic acid (C20:1*n*-9) in addition to CA^(1)^, therefore both fatty acids may serve as markers for dietary intake of fish such as herring. C20:1*n*-9 was recovered in higher amounts in liver CE, liver PL, blood cells, muscle and WAT in the HERO group, whereas the C20:1*n*-9 concentration was similar between the ANCO group and the control group for all analysed tissues (online Supplementary Tables 1–8). Saturated fatty acids can be delta-9 desaturated in many tissues, producing in particular C16:1*n*-7 and C18:1*n*-9 from C16:0 and C18:0, respectively. The relative concentrations of C16:1*n*-7 and C18:1*n*-9 were similar between all three groups in liver TAG, liver NEFA, muscle and WAT, whereas the amount of C16:1*n*-7 was higher in liver PL, blood cells and brain in the HERO group compared with controls.

### Desaturases in liver and skeletal muscle

The hepatic content of FADS2 was significantly higher in the ANCO group compared with the control group and the HERO group, with no differences between the groups for the hepatic concentrations of FADS1 and SCD1 (Table 3). The muscle contents of FADS2 and FADS1 were similar between all three groups.

**Table 3.** Contents of desaturases in the liver and in skeletal muscle (presented relative to protein content)

ng/mg protein	Control group		HERO group		ANCO group		*P* ANOVA
	Mean	sd	Mean	sd	Mean	sd	
Liver FADS2	28·4	8·3^a^	19·6	9·5^a^	45·7	11·1^b^	0·0010
Liver FADS1	201	24	221	22	234	28	0·097
Liver SCD1	597	109	583	52	685	126	0·20
Muscle FADS2	3·97	2·89	4·02	1·09	4·61	8·21	0·91
Muscle FADS1	1·59	3·13	1·42	0·82	1·54	1·32	0·97

HERO, herring oil; ANCO, anchovy oil; FADS2, fatty acid desaturase 2, FADS1, fatty acid desaturase 1; SCD1, stearoyl-CoA desaturase 1.

Data are presented as mean (standard deviation) for *n* 6 rats in each experimental group. Groups are compared using one-way ANOVA followed by Tukey HSD post hoc test when appropriate. Means in a row with different letters are significantly different (*P* < 0·05).

## Discussion

In this study, we show for the first time that when ZDSD rats are fed a diet containing herring oil, this results in the accumulation of *n*-11 MUFAs in liver lipids, blood cells, WATepi and skeletal muscle but not in brain tissue. We also show that the level of EPA was higher in liver lipids (PL, TAG, CE and NEFA) as well as in blood cells, WATepi, muscle and brain from rats fed the HERO diet when compared with the control group, with no differences between the HERO group and the ANCO group. The effects of herring oil intake were less systematic with regard to the DHA level in various liver lipids and extrahepatic tissues but was higher in liver CE and NEFA, similar to that of the control group in liver PL, TAG and higher in blood cells and in WATepi but was not affected in muscle and brain. The levels of EPA and DHA were similar between the HERO group and the ANCO group with regard to liver lipids with the exception of DHA in liver PL and all extrahepatic tissues with the exception of blood cells where the DHA level was higher in the HERO rats. The higher levels of EPA and DHA in rats fed the HERO diet compared with controls are probably not caused by an increased endogenous synthesis since the protein concentrations of FADS2 and FADS1 in liver and muscle were not different from those of the control group, and since EPA and DHA levels were comparable in the HERO diet and the ANCO diet.

Long-chain *n*-11 MUFA may be excellent biomarkers for compliance in clinical studies and animal studies when studying the effects of consumption of filets or oils from fish species such as herring. Herring contain relatively high amounts of CA since they feed on zooplankton such as copepods which produce 22:1*n*-11 *de novo* ^(1)^, whereas vertebrates have limited capacity to biosynthesise CA. However, recent research indicates that long-chain *n*-11 MUFAs such as CA also may have beneficial health effects, including lowering of cholesterol^(7,8)^ and improved glucose metabolism^(27)^ in rodents. Accumulation of exogenous fatty acids such as the long-chain *n*-11 MUFAs may induce functional changes in membrane composition thus affecting membrane fluidity and enzyme activities, either as direct effects or by affecting the amounts of other fatty acids through changes in catabolic and anabolic processes including desaturation of fatty acids, or affects metabolic pathways through other mechanisms of action. A comprehensive survey of the occurrence CA, GA and 7OA (and possibly other *n*-11 MUFAs) in various tissues and lipid classes have not been conducted previously; however, others have found CA in plasma^(28–30)^, WAT^(6,28–31)^, liver^(28–32)^, muscle^(30)^, plasma PL^(5)^ and liver PL^(5)^ in rodents fed diets containing long-chain MUFA concentrates^(5,6,28,31,32)^, pollock oil^(29)^ or saury oil^(30)^, and GA was found in plasma^(31)^, liver^(31,32)^ WAT^(6,31)^ after long-chain MUFA consumption^(6,31,32)^. Accumulation of 7OA was shown only in one article, that is, in WAT from rats fed a diet containing long-chain MUFA^(6)^. In line with this, the present comprehensive study demonstrates that CA and the shorter *n*-11 MUFAs GA and 7OA accumulate in liver lipids, blood cells, WATepi and muscle after intake of feed containing herring oil, but we found no *n*-11 MUFAs in the brain tissue. GA and 7OA were found in amounts that were comparable to, or higher, than CA in the tissues, and since the HERO diet contained very low amounts of GA and 7OA when compared with CA, it is likely that GA and 7OA are products of beta-oxidation of CA in the rats. We did not identify any *n*-11 MUFAs with < 18 carbons or > 22 carbons in any of the tissues that were analysed.

The knowledge about the health effects of CA and its *n*-11 MUFA metabolites is scarce. Based on findings in cell culture studies, it has been speculated that a beneficial effect of CA may be mediated through stimulation of the endogenous synthesis of EPA and DHA by upregulating the FADS1 and FADS2 mRNA levels in the liver^(3)^. Since most studies that explore the effects of CA have used fish oils, it is challenging to interpret if these oils induce higher synthesis of EPA and DHA since fish oils also contain long-chain *n*-3 PUFA, or if the higher EPA and DHA contents originate from the diet. A study in LDLr –/– mice using diets containing an EPA- and DHA-free CA concentrate did not find higher hepatic levels of EPA and DHA^(32)^, whereas several studies using CA-rich fish oil reported higher hepatic concentrations of EPA and/or DHA in rodents^(7)^. In this study, the EPA + DHA content was comparable between the HERO diet and the ANCO diet, and we found no difference between the HERO group and the ANCO group in the relative amounts of EPA and DHA in liver, WAT, muscle or brain. However, we found a higher relative amount of DHA in liver PL and blood cells as well as a higher absolute DHA amount in the liver in the HERO group when compared with the ANCO group, which may be a reflection of the marginally higher DHA content in the HERO diet compared with the ANCO diet. Since the hepatic FADS2 level was lower in HERO rats compared with ANCO rats, combined with similar hepatic FADS1 concentration and of FADS2 and FADS1 in muscle in these two groups, it is not likely that the endogenous production of EPA and DHA from *α*-linolenic acid was higher in rats in the HERO group. This is also supported by findings in another rat study, where the hepatic gene expressions of both FADS2 and FADS1 were lower in rats fed a diet containing sandeel oil compared with rats fed a control diet with soyabean oil^(33)^. The higher FADS2 concentration (catalysing the delta-6 desaturation of 18:2*n*-6 to 18:3*n*-6, and of 18:3*n*-3 to 18:4*n*-3) in livers from rats fed the ANCO diet compared with those fed the HERO diet was not reflected in higher amounts of any of the desaturated and elongated products of 18:2*n*-6 and 18:3*n*-3 in any of the tissues investigated.

We recently presented data indicating that neither HERO nor ANCO diets affected the *de novo* lipogenesis from glucose, the TAG synthesis or the rate of VLDL assembly in the liver from ZDSD rats^(8)^. Here, we show that in the same rats, SCD1 was not affected by intake of HERO or ANCO diets, supporting the previous finding that the hepatic fatty acid synthesis was not affected by these diets. In line with this, the relative concentrations of the delta-9 desaturated MUFAs C16:1*n*-7 and C18:1*n*-9 from both groups were similar to controls in liver TAG, liver NEFA, muscle and WAT. The higher level of C16:1*n*-7 in BC and liver PL from rats fed the HERO or the ANCO diets may be a reflection of the higher content of this fatty acid in both the HERO diet and the ANCO diet compared to the control diet, rather than a result of increased endogenous synthesis. The HERO diet also contained a considerable amount of C20:1*n*-9, originating from copepods consumed by the herring^(1)^, and correspondingly, rats fed the HERO diet had a higher concentration of C20:1*n*-9 in BC, liver CE, liver PL, muscle and WAT. The ANCO diet and the control diet contained very little C20:1n-9, and the C20:1*n*-9 concentration was similar to controls in rats fed the ANCO diet in all investigated tissues. These findings support the assumption that the higher levels of C16:1*n*-7, C18:1*n*-9 and C20:1*n*-9 results from dietary intake and not endogenous production.

The present study presents evidence that CA from the HERO diet is absorbed from the diet and is accumulated and metabolised in tissues with diverse structure and function in blood cells, liver, WATepi and skeletal muscle, but we did not find any form of *n*-11 MUFAs in the brain. Although the present findings in this rat study are not directly transferable to humans, the mechanisms for uptake and storage as well as the metabolism of CA might be similar to those in other animals such as humans and should be further investigated in clinical studies.

This study has some strengths and limitations. Strengths include the quantification of fatty acids in a range of tissues with different characteristics and functions. The presence of CA and its shorter metabolites GA and 7OA in liver, blood cells, WAT and muscle indicate that these *n*-11 MUFAs are integrated in cells and thus may affect biochemical and physiological processes. Limitations to the study include the choice of animal model used, as the observed effects of fish oil intake on CA uptake and desaturases in rats with overt type 2 diabetes may be specific to the ZDSD rats since diabetes and obesity influence the gene expressions and activities of the desaturases in the liver of rats^(34,35)^. In this study, we measured the amounts and not the activities of the desaturases, and since the elongases involved in the LC-PUFA synthesis, that is, ELOVL2 and ELOVL5, were not quantified, we cannot with absolute certainty conclude that CA did not affect the endogenous synthesis of EPA and DHA. The biological availability of CA from the HERO may be specific to this particular fish oil and cannot be generalised to other fish oils containing CA; hence, other CA-rich fish oils should be investigated in both human and animal models.

### Conclusion

In this study, we present evidence that CA from herring oil is absorbed, accumulated and metabolised in tissues with diverse structure and function; in blood cells, liver, WATepi and skeletal muscle but not in the brain. In contrast, no *n*-11 MUFAs were found in the investigated tissues harvested from the control group or in the ANCO group. Thus, *n*-11 MUFAs may be used as biomarkers for compliance in intervention studies with the consumption of CA-rich fish or fish oil from species such as herring. More importantly, the accumulation of *n*-11 MUFAs may induce functional changes in membrane composition thus affecting membrane fluidity and enzyme activities, either as direct effects or by affecting the amounts of other fatty acids or through changes in catabolic and anabolic processes including desaturation of fatty acids. The dietary content of EPA + DHA was similar between the HERO diet and the ANCO diet, and we show that the concentrations of EPA and DHA were similar in liver (with the exception of DHA in liver PL), WATepi, muscle and brain from rats fed the HERO diet or the ANCO diet. This, combined with the similar FADS2 and FADS1 concentrations in the liver and muscle between rats fed the HERO diet and the control diet indicates that CA does not stimulate the endogenous synthesis of EPA and DHA in this diabetic rat model.

## Supporting information

Rimmen et al. supplementary material 1 Rimmen et al. supplementary material

Rimmen et al. supplementary material 2 Rimmen et al. supplementary material

## Supplementary material

For supplementary material/s referred to in this article, please visit https://doi.org/10.1017/S0007114525000236
