# Supplementary material for: Absorption, accumulation and metabolism of cetoleic acid from dietary herring oil in tissues of male Zucker Diabetic Sprague Dawley rats
Source: Br J Nutr. 2025 Feb 14;133(5):577–85. doi: 10.1017/S0007114525000236 (PMC12055446; doi:10.1017/S0007114525000236)
Supplement: Rimmen et al. supplementary material 1 — Rimmen et al. supplementary material [file S0007114525000236sup001.pptx]

## Slide 1
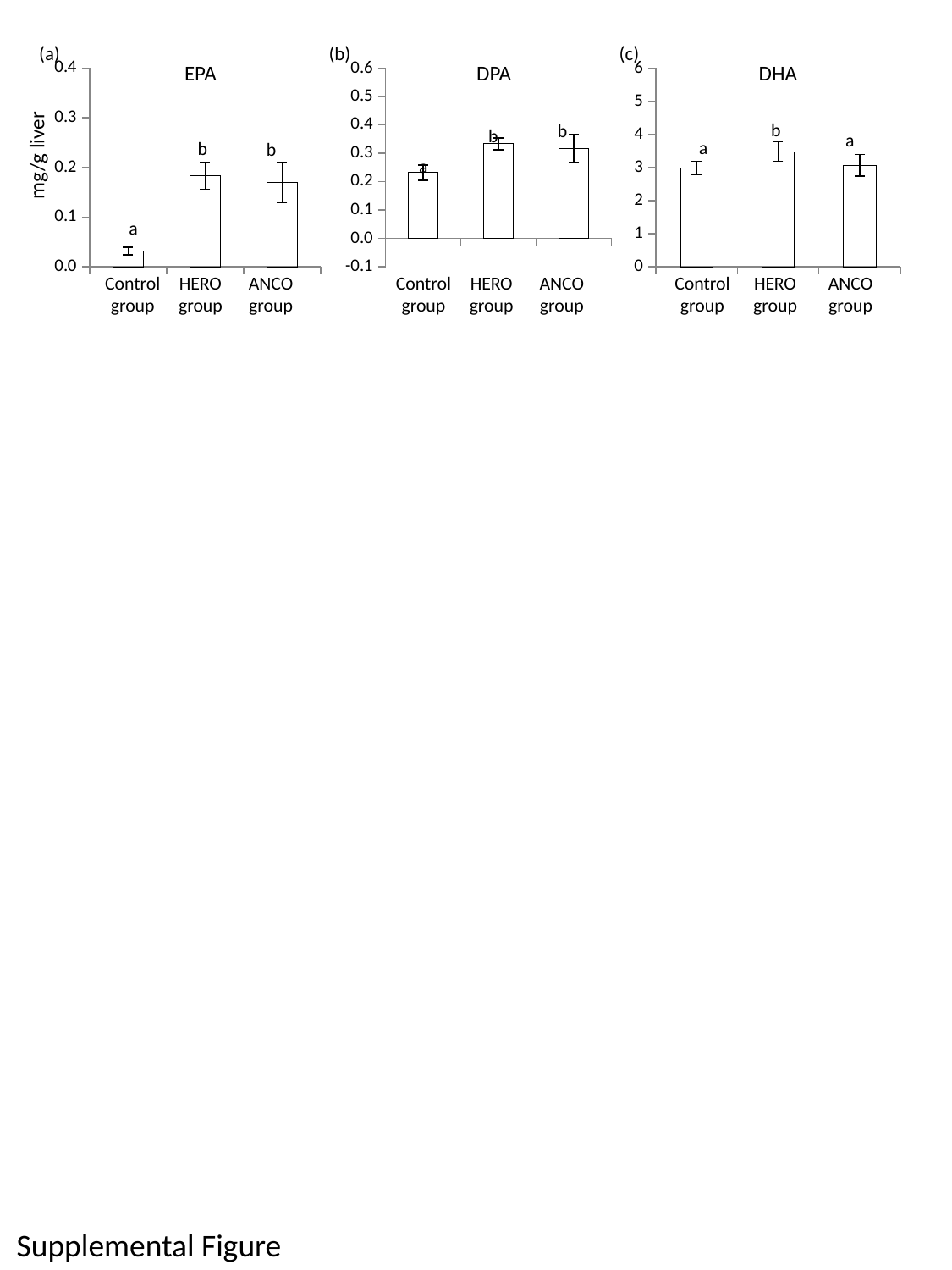

(b)
(a)
(c)
EPA
DPA
DHA
### Chart
| Category | |
|---|---|
| CTRL | 0.031742193509595416 |
| HERO | 0.1833193077116031 |
| ANCO | 0.17001304277502757 |
### Chart
| Category | |
|---|---|
| CTRL | 0.23139235382891635 |
| HERO | 0.3330192485227017 |
| ANCO | 0.31791079876465583 |
### Chart
| Category | |
|---|---|
| CTRL | 2.9905917551211467 |
| HERO | 3.477834017798974 |
| ANCO | 3.0643474722677433 |b
b
b
a
a
b
b
mg/g liver
a
a
HERO
group
ANCO
group
HERO
group
ANCO
group
HERO
group
ANCO
group
Control
group
Control
group
Control
group
Supplemental Figure
