# Supplementary material for: Absorption, accumulation and metabolism of cetoleic acid from dietary herring oil in tissues of male Zucker Diabetic Sprague Dawley rats
Source: Br J Nutr. 2025 Feb 14;133(5):577–85. doi: 10.1017/S0007114525000236 (PMC12055446; doi:10.1017/S0007114525000236)
Supplement: Rimmen et al. supplementary material 2 — Rimmen et al. supplementary material [file S0007114525000236sup002.docx]

**Supplemental table 1**: Fatty acids in liver phospholipids, presented as g/100g fatty acids (mean values and standard deviations)

|  | Control group | HERO group | ANCO group | *P* ANOVA |
| --- | --- | --- | --- | --- |
| C14:0 | 0.043 ± 0.003^a^ | 0.064 ± 0.007^b^ | 0.052 ± 0.009^c^ | 1.1x10^-4^ |
| C15:0 | 0.063 ± 0.002^a^ | 0.088 ± 0.008^b^ | 0.077 ± 0.003^c^ | 2.3x10^-6^ |
| C16:0 | 13.39 ± 0.252^a^ | 14.594 ± 0.546^b^ | 14.505 ± 0.37^b^ | 1.8x10^-4^ |
| C17:0 | 0.344 ± 0.007^a^ | 0.388 ± 0.013^b^ | 0.389 ± 0.010^b^ | 1.7x10^-6^ |
| C18:0 | 26.831 ± 0.174^a^ | 25.133 ± 0.751^b^ | 25.602 ± 0.722^b^ | 6.6x10^-4^ |
| C20:0 | 0.129 ± 0.006^a^ | 0.120 ± 0.010^ab^ | 0.116 ± 0.007^b^ | 0.039 |
| C22:0 | 0.460 ± 0.027^a^ | 0.256 ± 0.012^b^ | 0.372 ± 0.029^c^ | 1.3x10^-9^ |
| C23:0 | 0.456 ± 0.031^a^ | 0.310 ± 0.025^b^ | 0.410 ± 0.033^c^ | 1.4x10^-6^ |
| C24:0 | 0.914 ± 0.043^a^ | 0.469 ± 0.017^b^ | 0.788 ± 0.033^c^ | 9.2x10^-13^ |
| C16:1 n-7 | 0.055 ± 0.007^a^ | 0.108 ± 0.013^b^ | 0.110 ± 0.043^b^ | 3.3x10^-3^ |
| C16:1 n-9 | 0.075 ± 0.006^a^ | 0.093 ± 0.012^b^ | 0.085 ± 0.011^ab^ | 0.026 |
| C18:1 n-7 | 1.501 ± 0.088 | 1.488 ± 0.062 | 1.602 ± 0.193 | 0.27 |
| C18:1 n-9 | 2.654 ± 0.216 | 2.938 ± 0.222 | 2.890 ± 0.224 | 0.089 |
| C20:1 n-7 | 0.037 ± 0.011 | 0.038 ± 0.009 | 0.036 ± 0.008 | 0.87 |
| C20:1 n-9 | 0.079 ± 0.010^a^ | 0.475 ± 0.058^b^ | 0.085 ± 0.014^a^ | 2.7x10^-12^ |
| C22:1 n-9 | 0.019 ± 0.016 | 0.030 ± 0.015 | 0.013 ± 0.011 | 0.15 |
| C24:1 n-9 | 0.113 ± 0.012^a^ | 0.647 ± 0.037^b^ | 0.214 ± 0.008^c^ | 2.0x10­^-16^ |
| C18:1 n-11 | <LOQ | 0.237 ± 0.052 | <LOQ |  |
| C20:1 n-11 | <LOQ | 0.120 ± 0.018 | <LOQ |  |
| C22:1 n-11 | <LOQ | 0.244 ± 0.020 | <LOQ |  |
| C18:2 n-6 | 12.103 ± 0.672^a^ | 12.765 ± 0.351^ab^ | 13.523 ± 0.776^b^ | 4.9x10^-3^ |
| C18:3 n-6 | 0.153 ± 0.012 | 0.147 ± 0.020 | 0.147 ± 0.007 | 0.72 |
| C20:3 n-6 | 0.737 ± 0.058^a^ | 1.350 ± 0.127^b^ | 1.338 ± 0.113^b^ | 2.8x10^-8^ |
| C20:4 n-6 | 27.136 ± 0.669^a^ | 23.460 ± 0.679^b^ | 24.548 ± 1.369^b^ | 2.6x10^-5^ |
| C22:4 n-6 | 0.433 ± 0.021^a^ | 0.191 ± 0.010^b^ | 0.219 ± 0.019^c^ | 2.9x10^-13^ |
| C22:5 n-6 | 0.217 ± 0.038^a^ | 0.055 ± 0.005^b^ | 0.056 ± 0.008^b^ | 1.7x10^-9^ |
| C18:3 n-3 | 0.101 ± 0.017^a^ | 0.070 ± 0.008^b^ | 0.080 ± 0.013^b^ | 3.7x10^-3^ |
| C20:5 n-3 | 0.078 ± 0.015^a^ | 0.560 ± 0.097^b^ | 0.500 ± 0.107^b^ | 7.9x10^-8^ |
| C22:5 n-3 | 0.776 ± 0.074^a^ | 1.017 ± 0.103^b^ | 0.924 ± 0.103^b^ | 1.7x10^-3^ |
| C22:6 n-3 | 11.094 ± 0.337^a^ | 12.516 ± 0.514^b^ | 11.309 ± 0.555^a^ | 2.3x10^-4^ |

HERO, herring oil; ANCO, anchovy oil.

Data are presented as mean and standard deviation for *n* 6 rats in each experimental group. Groups are compared using one-way ANOVA followed by Tukey HSD post hoc test when appropriate. Means in a row with different letters are significantly different (*P* < 0.05). LOQ, level of quantification

**Supplemental table 2**: Fatty acids in liver triacylglycerols, presented as g/100g fatty acids (mean values and standard deviations)

|  | Control group | HERO group | ANCO group | *P* ANOVA |
| --- | --- | --- | --- | --- |
| C12:0 | 2.456 ± 1.674 | 2.544 ± 2.407 | 0.582 ± 0.484 | 0.12 |
| C14:0 | 1.395 ± 0.547^a^ | 1.249 ± 0.453^ab^ | 0.629 ± 0.355^b^ | 0.026 |
| C15:0 | 0.509 ± 0.292 | 0.593 ± 0.453 | 0.202 ± 0.031 | 0.11 |
| C16:0 | 29.030 ± 4.922 | 28.490 ± 5.535 | 26.119 ± 4.583 | 0.58 |
| C17:0 | 0.474 ± 0.131 | 0.478 ± 0.154 | 0.371 ± 0.090 | 0.29 |
| C18:0 | 14.617 ± 4.472 | 10.747 ± 4.265 | 9.36 ± 5.099 | 0.16 |
| C20:0 | 2.029 ± 1.181 | 2.435 ± 2.098 | 0.798 ± 0.613 | 0.16 |
| C22:0 | 3.146 ± 2.195 | 3.766 ± 3.590 | 0.984 ± 0.851 | 0.16 |
| C24:0 | 0.371 ± 0.334 | 0.174 ± 0.110 | 0.224 ± 0.177 | 0.33 |
| C16:1 n-7 | 0.954 ± 1.027 | 1.119 ± 0.828 | 1.430 ± 1.672 | 0.80 |
| C16:1 n-9 | 0.421 ± 0.190 | 0.487 ± 0.204 | 0.358 ± 0.157 | 0.50 |
| C18:1 n-7 | 1.827 ± 0.865 | 1.648 ± 0.877 | 2.722 ± 0.468 | 0.059 |
| C18:1 n-9 | 14.915 ± 11.428 | 16.557 ± 11.470 | 29.337 ± 3.840 | 0.039 |
| C20:1 n-7 | 1.332 ± 1.139 | 1.003 ± 0.750 | 0.386 ± 0.200 | 0.15 |
| C22:1 n-9 | 0.048 ± 0.040^a^ | 0.207 ± 0.071^b^ | 0.061 ± 0.028^a^ | 1.4x10^-4^ |
| C24:1 n-9 | 0.092 ± 0.059^ab^ | 0.164 ± 0.071^a^ | 0.059 ± 0.033^b^ | 0.017 |
| C18:1 n-11 | <LOQ | 1.141 ± 0.487 | <LOQ |  |
| C20:1 n-11 | <LOQ | 1.637 ± 0.769 | <LOQ |  |
| C22:1 n-11 | <LOQ | 3.548 ± 1.636 | <LOQ |  |
| C18:2 n-6 | 14.144 ± 3.771 | 13.930 ± 4.682 | 17.977 ± 9.029 | 0.47 |
| C18:3 n-6 | 0.155 ± 0.062 | 0.126 ± 0.049 | 0.154 ± 0.119 | 0.79 |
| C20:3 n-6 | 0.652 ± 0.323 | 0.593 ± 0.411 | 0.887 ± 0.573 | 0.50 |
| C20:4 n-6 | 6.404 ± 3.171^a^ | 2.726 ± 0.889^b^ | 2.971 ± 1.854^b^ | 0.018 |
| C22:4 n-6 | 0.950 ± 0.260^a^ | 0.345 ± 0.158 ^b^ | 0.451 ± 0.325 ^b^ | 0.0022 |
| C22:5 n-6 | 0.175 ± 0.103 | 0.057 ± 0.054 | 0.092 ± 0.079 | 0.061 |
| C18:3 n-3 | 0.292 ± 0.123 | 0.210 ± 0.067 | 0.230 ± 0.154 | 0.48 |
| C20:5 n-3 | 0.163 ± 0.064^a^ | 0.364 ± 0.262^b^ | 0.291 ± 0.081^ab^ | 0.024 |
| C22:5 n-3 | 0.502 ± 0.121 | 0.861 ± 0.386 | 0.744 ± 0.500 | 0.26 |
| C22:6 n-3 | 2.141 ± 1.095 | 2.695 ± 1.313 | 2.286 ± 1.145 | 0.71 |

HERO, herring oil; ANCO, anchovy oil.

Data are presented as mean and standard deviation for *n* 6 rats in each experimental group. Groups are compared using one-way ANOVA followed by Tukey HSD post hoc test when appropriate. Means in a row with different letters are significantly different (*P* < 0.05). LOQ, level of quantification

**Supplemental table 3**: Fatty acids in liver cholesteryl esters, presented as g/100g fatty acids (mean values and standard deviations)

|  | Control group | HERO group | ANCO group | *P* ANOVA |
| --- | --- | --- | --- | --- |
| C12:0 | 2.316 ± 0.944^a^ | 1.274 ± 0.221^b^ | 1.588 ± 0.535^ab^ | 0.036 |
| C14:0 | 0.309 ± 0.159 | 0.295 ± 0.053 | 0.307 ± 0.076 | 0.97 |
| C15:0 | 0.308 ± 0.123 | 0.203 ± 0.038 | 0.235 ± 0.051 | 0.095 |
| C16:0 | 34.160 ± 3.568^a^ | 26.453 ± 5.263^b^ | 25.838 ± 5.335^b^ | 0.015 |
| C17:0 | 0.562 ± 0.080 | 0.534 ± 0.124 | 0.465 ± 0.127 | 0.34 |
| C18:0 | 9.789 ± 0.601^a^ | 6.922 ± 1.526^b^ | 6.663 ± 1.770^b^ | 2.4x10^-3^ |
| C23:0 | 0.220 ± 0.052^a^ | 0.173 ± 0.049^ab^ | 0.133 ± 0.038^b^ | 0.027 |
| C16:1 n-7 | 1.039 ± 0.607 | 1.156 ± 0.245 | 1.448 ± 0.319 | 0.25 |
| C16:1 n-9 | 0.291 ± 0.172 | 0.206 ± 0.028 | 0.226 ± 0.080 | 0.41 |
| C18:1 n-7 | 1.080 ± 0.149^a^ | 1.324 ± 0.091^ab^ | 1.308 ± 0.144^b^ | 8.9x10^-3^ |
| C18:1 n-9 | 15.530 ± 2.645^a^ | 25.352 ± 3.329^b^ | 24.131 ± 4.655^b^ | 5.3x10^-4^ |
| C20:1 n-9 | <LOQ | 0.624 ± 0.057 | <LOQ |  |
| C18:1 n-11 | <LOQ | 0.597 ± 0.096 | <LOQ |  |
| C20:1 n-11 | <LOQ | 0.498 ± 0.079 | <LOQ |  |
| C22:1 n-11 | <LOQ | 0.825 ± 0.065 | <LOQ |  |
| C18:2 n-6 | 16.801 ± 2.540^a^ | 19.887 ± 3.589^a^ | 22.434 ± 4.158^b^ | 0.043 |
| C18:3 n-6 | 0.432 ± 0.103 | 0.330 ± 0.059 | 0.391 ± 0.046 | 0.083 |
| C20:3 n-6 | 0.351 ± 0.027 | 0.379 ± 0.055 | 0.406 ± 0.037 | 0.13 |
| C20:4 n-6 | 13.451 ± 1.120^a^ | 8.510 ± 0.649^b^ | 9.541 ± 1.095^b^ | 6.6x10^-7^ |
| C18:3 n-3 | 1.055 ± 0.297 | 1.195 ± 0.440 | 1.514 ± 0.344 | 0.12 |
| C20:5 n-3 | 0.145 ± 0.073^a^ | 0.995 ± 0.177^b^ | 1.134 ± 0.182^b^ | 1.7x10^-8^ |
| C22:6 n-3 | 1.342 ± 0.119^a^ | 2.096 ± 0.391^b^ | 1.741 ± 0.292^ab^ | 1.6x10^-3^ |

HERO, herring oil; ANCO, anchovy oil.

Data are presented as mean and standard deviation for *n* 6 rats in each experimental group. Groups are compared using one-way ANOVA followed by Tukey HSD post hoc test when appropriate. Means in a row with different letters are significantly different (*P* < 0.05). LOQ, level of quantification

**Supplemental table 4**: Fatty acids in liver free fatty acids, presented as g/100g fatty acids (mean values and standard deviations)

|  | Control group | HERO group | ANCO group | *P* ANOVA |
| --- | --- | --- | --- | --- |
| C12:0 | 1.733 ± 1.085 | 1.611 ± 0.946 | 2.140 ± 1.481 | 0.73 |
| C14:0 | 0.987 ± 0.716 | 0.800 ± 0.333 | 0.826 ± 0.427 | 0.80 |
| C15:0 | 0.330 ± 0.141 | 0.284 ± 0.121 | 0.284 ± 0.124 | 0.80 |
| C16:0 | 20.320 ± 11.347 | 13.909 ± 9.888 | 14.436 ± 5.767 | 0.44 |
| C17:0 | 0.561 ± 0.254 | 0.393 ± 0.218 | 0.387 ± 0.107 | 0.27 |
| C18:0 | 11.609 ± 7.396 | 7.324 ± 6.548 | 8.667 ± 6.001 | 0.54 |
| C14:1 n-5 | 2.592 ± 1.717 | 2.016 ± 1.245 | 2.188 ± 1.657 | 0.81 |
| C16:1 n-7 | 0.488 ± 0.291 | 0.584 ± 0.285 | 0.350 ± 0.117 | 0.28 |
| C16:1 n-9 | 0.325 ± 0.040 | 0.372 ± 0.092 | 0.344 ± 0.162 | 0.79 |
| C18:1 n-7 | 0.828 ± 0.228 | 0.786 ± 0.227 | 0.722 ± 0.344 | 0.80 |
| C18:1 n-9 | 4.921 ± 1.327 | 5.220 ± 1.450 | 4.433 ± 0.807 | 0.55 |
| C20:1 n-9 | 0.351 ± 0.252 | 0.439 ± 0.245 | 0.232 ± 0.251 | 0.37 |
| C18:1 n-11 | <LOQ | 0.400 ± 0.178 | <LOQ |  |
| C20:1 n-11 | <LOQ | 0.235 ± 0.125 | <LOQ |  |
| C22:1 n-11 | <LOQ | 0.346 ± 0.272 | <LOQ |  |
| C18:2 n-6 | 20.285 ± 9.461 | 21.023 ± 6.939 | 22.297 ± 6.728 | 0.90 |
| C20:3 n-6 | 1.215 ± 0.658 | 0.954 ± 0.324 | 0.996 ± 0.482 | 0.64 |
| C20:4 n-6 | 8.165 ± 5.529 | 4.333 ± 3.218 | 4.788 ± 2.900 | 0.21 |
| C22:4 n-6 | 1.547 ±1.093^a^ | 0.422 ± 0.165^b^ | 0.376 ± 0.173^b^ | 0.010 |
| C18:3 n-3 | 2.217 ± 0.916 | 2.484 ± 1.936 | 3.424 ± 1.323 | 0.31 |
| C20:5 n-3 | 1.461 ± 0.452^a^ | 3.346 ± 1.146^b^ | 3.590 ± 0.920^b^ | 1.5x10^-3^ |
| C22:5 n-3 | 3.972 ± 1.654^a^ | 7.618 ± 2.792^b^ | 6.831 ± 2.574^ab^ | 0.044 |
| C22:6 n-3 | 12.578 ± 4.792^a^ | 23.776 ± 8.811^b^ | 20.492 ± 6.298^ab^ | 0.034 |

HERO, herring oil; ANCO, anchovy oil.

Data are presented as mean and standard deviation for *n* 6 rats in each experimental group. Groups are compared using one-way ANOVA followed by Tukey HSD post hoc test when appropriate. Means in a row with different letters are significantly different (*P* < 0.05). LOQ, level of quantification

**Supplemental table 5**: Fatty acids in blood cells, presented as g/100g fatty acids (mean values and standard deviations)

|  | Control group | HERO group | ANCO group | *P* ANOVA |
| --- | --- | --- | --- | --- |
| C14:0 | 0.181 ± 0.020^a^ | 0.357 ± 0.045^b^ | 0.238 ± 0.014^c^ | 1.2x10^-7^ |
| C15:0 | 0.192 ± 0.013^a^ | 0.264 ± 0.024^b^ | 0.220 ± 0.018^a^ | 2.2x10^-5^ |
| C16:0 | 20.340 ± 0.627^a^ | 21.649 ± 0.568^b^ | 21.522 ± 0.508^b^ | 2.0x10^-3^ |
| C17:0 | 0.210 ± 0.009^a^ | 0.246 ± 0.027^b^ | 0.252 ± 0.023^b^ | 2.9x10^-3^ |
| C18:0 | 6.006 ± 0.794 | 6.688 ± 1.688 | 7.215 ± 1.395 | 0.24 |
| C22:0 | 0.275 ± 0.126 | 0.223 ± 0.163 | 0.375 ± 0.129 | 0.15 |
| C24:0 | 1.044 ± 0.475 | 0.823 ± 0.613 | 1.491 ± 0.413 | 0.084 |
| C16:1 n-7 | 0.101 ± 0.018^a^ | 0.215 ± 0.021^b^ | 0.194 ± 0.041^b^ | 1.2x10^-5^ |
| C16:1 n-9 | 0.139 ± 0.017 | 0.137 ± 0.008 | 0.134 ± 0.018 | 0.84 |
| C18:1 n-7 | 1.092 ± 0.072 | 1.151 ± 0.098 | 1.203 ± 0.083 | 0.11 |
| C18:1 n-9 | 3.917 ± 0.262^a^ | 4.425 ± 0.292^b^ | 4.340 ± 0.335^ab^ | 0.022 |
| C20:1 n-9 | 0.030 ± 0.006^a^ | 0.147 ± 0.029^b^ | 0.028 ± 0.007^a^ | 5.9x10^-9^ |
| C22:1 n-9 | <LOQ | 0.011 ± 0.003 | <LOQ |  |
| C24:1 n-9 | 0.345 ± 0.189 | 0.659 ± 0.443 | 0.588 ± 0.245 | 0.084 |
| C18:1 n-11 | <LOQ | 0.157 ± 0.009 | <LOQ |  |
| C20:1 n-11 | <LOQ | 0.029 ± 0.008 | <LOQ |  |
| C22:1 n-11 | <LOQ | 0.072 ± 0.037 | <LOQ |  |
| C18:2 n-6 | 15.603 ± 0.635 | 15.303 ± 1.006 | 15.133 ± 1.109 | 0.69 |
| C18:3 n-6 | 0.159 ± 0.041^a^ | 0.111 ± 0.010^b^ | 0.127 ± 0.024^ab^ | 0.029 |
| C20:3 n-6 | 0.562 ± 0.042^a^ | 0.680 ± 0.023^b^ | 0.675 ± 0.052^b^ | 1.8x10^-4^ |
| C20:4 n-6 | 40.451 ± 1.403^a^ | 34.501 ± 1.532^b^ | 35.142 ± 1.700^b^ | 2.4x10^-5^ |
| C22:4 n-6 | 0.921 ± 0.090^a^ | 0.608 ± 0.104^b^ | 0.697 ± 0.073^b^ | 7.2x10^-5^ |
| C22:5 n-6 | 0.419 ± 0.055^a^ | 0.249 ± 0.056^b^ | 0.309 ± 0.049^b^ | 2.0x10^-4^ |
| C18:3 n-3 | 0.261 ± 0.052^a^ | 0.183 ± 0.027^b^ | 0.183 ± 0.026^b^ | 2.9x10^-3^ |
| C20:5 n-3 | 0.337 ± 0.026^a^ | 2.084 ± 0.413^b^ | 1.834 ± 0.201^b^ | 1.4x10^-8^ |
| C22:5 n-3 | 1.649 ± 0.120^a^ | 1.909 ± 0.105^b^ | 1.869 ± 0.128^b^ | 3.6x10^-3^ |
| C22:6 n-3 | 5.538 ± 0.253^a^ | 6.719 ± 0.652^b^ | 5.944 ± 0.238^a^ | 8.1x10^-4^ |

HERO, herring oil; ANCO, anchovy oil.

Data are presented as mean and standard deviation for *n* 6 rats in each experimental group. Groups are compared using one-way ANOVA followed by Tukey HSD post hoc test when appropriate. Means in a row with different letters are significantly different (*P* < 0.05). LOQ, level of quantification

**Supplemental table 6**: Fatty acids in epididymal white adipose tissue, presented as g/100g fatty acids (mean values and standard deviations)

|  | Control group | HERO group | ANCO group | *P* ANOVA |
| --- | --- | --- | --- | --- |
| C12:0 | 0.048 ± 0.005 | 0.051 ± 0.002 | 0.047 ± 0.006 | 0.42 |
| C14:0 | 0.811 ± 0.060^a^ | 0.927 ± 0.053^b^ | 0.832 ± 0.089^ab^ | 0.025 |
| C15:0 | 0.167 ± 0.016 | 0.188 ± 0.009 | 0.174 ± 0.022 | 0.11 |
| C16:0 | 20.590 ± 0.642 | 20.943 ± 0.673 | 20.604 ± 1.096 | 0.71 |
| C17:0 | 0.277 ± 0.012 | 0.278 ± 0.007 | 0.295 ± 0.017 | 0.047 |
| C18:0 | 4.497 ± 0.159 | 4.267 ± 0.190 | 4.616 ± 0.317 | 0.057 |
| C14:1 n-5 | 0.031 ± 0.005 | 0.035 ± 0.005 | 0.026 ± 0.010 | 0.13 |
| C16:1 n-7 | 1.928 ± 0.257 | 2.125 ± 0.235 | 1.802 ± 0.476 | 0.28 |
| C16:1 n-9 | 0.499 ± 0.027 | 0.502 ± 0.011 | 0.528 ± 0.036 | 0.16 |
| C17:1 n-8 | 0.135 ± 0.008 | 0.142 ± 0.007 | 0.131 ± 0.012 | 0.14 |
| C18:1 n-7 | 3.595 ± 0.208 | 3.505 ± 0.137 | 3.640 ± 0.265 | 0.54 |
| C18:1 n-9 | 36.971 ± 1.56 | 36.506 ± 0.725 | 37.822 ± 1.802 | 0.30 |
| C20:1 n-9 | 0.426 ± 0.021^a^ | 0.595 ± 0.059^b^ | 0.448 ± 0.036^a^ | 7.6x10^-6^ |
| C18:1 n-11 | <LOQ | 0.581 ± 0.065 | <LOQ |  |
| C20:1 n-11 | <LOQ | 0.320 ± 0.063 | <LOQ |  |
| C22:1 n-11 | <LOQ | 0.381 ± 0.152 | <LOQ |  |
| C18:2 n-6 | 27.946 ± 1.012 | 26.716 ± 0.372 | 27.087 ± 1.256 | 0.11 |
| C20:2 n-6 | 0.238 ± 0.019 | 0.231 ± 0.025 | 0.24 ± 0.023 | 0.77 |
| C20:3 n-6 | 0.137 ± 0.017^a^ | 0.100 ± 0.013^b^ | 0.121 ± 0.015^ab^ | 2.6x10^-3^ |
| C20:4 n-6 | 0.287 ± 0.035^a^ | 0.208 ± 0.027^b^ | 0.229 ± 0.012^b^ | 3.3x10^-4^ |
| C22:4 n-6 | 0.119 ± 0.013^a^ | 0.087 ± 0.021^b^ | 0.098 ± 0.022^ab^ | 0.031 |
| C18:3 n-3 | 1.053 ± 0.104 | 0.988 ± 0.043 | 0.948 ± 0.097 | 0.14 |
| C20:5 n-3 | 0.008 ± 0.007^a^ | 0.045 ± 0.009^b^ | 0.050 ± 0.015^b^ | 1.1x10^-5^ |
| C22:5 n-3 | 0.067 ± 0.007^a^ | 0.076 ± 0.020^ab^ | 0.096 ± 0.016^b^ | 7.3x10^-3^ |
| C22:6 n-3 | 0.088 ± 0.016^a^ | 0.129 ± 0.044^b^ | 0.131 ± 0.030^b^ | 0.015 |

HERO, herring oil; ANCO, anchovy oil.

Data are presented as mean and standard deviation for *n* 6 rats in each experimental group. Groups are compared using one-way ANOVA followed by Tukey HSD post hoc test when appropriate. Means in a row with different letters are significantly different (*P* < 0.05). LOQ, level of quantification

**Supplemental table 7**: Fatty acids in skeletal muscle from the thigh, presented as g/100g fatty acids (mean values and standard deviations)

|  | Control group | HERO group | ANCO group | *P* ANOVA |
| --- | --- | --- | --- | --- |
| C12:0 | 0.043 ± 0.008 | 0.048 ± 0.032 | 0.045 ± 0.035 | 0.95 |
| C14:0 | 0.355 ± 0.054 | 0.405 ± 0.121 | 0.527 ± 0.212 | 0.14 |
| C15:0 | 0.072 ± 0.012^a^ | 0.118 ± 0.018^b^ | 0.108 ± 0.009^b^ | 5.7x10^-5^ |
| C16:0 | 18.448 ± 3.342 | 21.2 ± 2.625 | 21.521 ± 4.357 | 0.28 |
| C17:0 | 0.374 ± 0.019^a^ | 0.425 ± 0.030^b^ | 0.397 ± 0.036^ab^ | 0.026 |
| C18:0 | 17.125 ± 2.682^a^ | 13.228 ± 2.183^b^ | 14.328 ± 2.597^ab^ | 0.044 |
| C20:0 | 0.261 ± 0.064 | 0.198 ± 0.069 | 0.196 ± 0.069 | 0.20 |
| C22:0 | 0.307 ± 0.085 | 0.218 ± 0.098 | 0.219 ± 0.054 | 0.13 |
| C23:0 | 0.415 ± 0.048 | 0.446 ± 0.129 | 0.411 ± 0.189 | 0.88 |
| C24:0 | 0.230 ± 0.143 | 0.118 ± 0.091 | 0.156 ± 0.043 | 0.19 |
| C16:1 n-7 | 0.948 ± 0.281 | 1.045 ± 0.492 | 1.344 ± 0.666 | 0.39 |
| C16:1 n-9 | 0.185 ± 0.031 | 0.167 ± 0.059 | 0.196 ± 0.038 | 0.54 |
| C18:1 n-7 | 1.801 ± 0.312 | 2.282 ± 0.442 | 2.242 ± 0.328 | 0.070 |
| C18:1 n-9 | 9.948 ± 1.043 | 10.258 ± 4.098 | 10.617 ± 2.174 | 0.92 |
| C20:1 n-9 | 0.213 ± 0.059^a^ | 0.775 ± 0.206^b^ | 0.315 ± 0.144^a^ | 2.0x10^-5^ |
| C22:1 n-9 | 0.342 ± 0.307 | 0.345 ± 0.207 | 0.326 ± 0.331 | 0.99 |
| C18:1 n-11 | <LOQ | 0.687 ± 0.317 | <LOQ |  |
| C20:1 n-11 | <LOQ | 0.336 ± 0.176 | <LOQ |  |
| C22:1 n-11 | <LOQ | 0.543 ± 0.240 | <LOQ |  |
| C18:2 n-6 | 27.576 ± 3.153 | 25.464 ± 4.322 | 26.105 ± 3.095 | 0.59 |
| C20:3 n-6 | 0.679 ± 0.126 | 0.769 ± 0.155 | 0.747 ± 0.084 | 0.45 |
| C20:4 n-6 | 12.000 ± 1.140^a^ | 8.533 ± 1.791^b^ | 9.061 ± 1.530^b^ | 0.0025 |
| C22:4 n-6 | 0.535 ± 0.052^a^ | 0.177 ± 0.050^b^ | 0.243 ± 0.057^b^ | 1.4x10^-8^ |
| C22:5 n-6 | 0.201 ± 0.040^a^ | 0.060 ± 0.034^b^ | 0.096 ± 0.047^b^ | 6.7x10^-5^ |
| C18:3 n-3 | 0.410 ± 0.158 | 0.345 ± 0.290 | 0.304 ± 0.076 | 0.65 |
| C18:4 n-3 | 0.021 ± 0.008 | 0.020 ± 0.007 | 0.020 ± 0.010 | 0.97 |
| C20:5 n-3 | 0.054 ± 0.008^a^ | 0.250 ± 0.038^b^ | 0.275 ± 0.060^b^ | 1.7x10^-7^ |
| C22:5 n-3 | 1.298 ± 0.387 | 1.644 ± 0.398 | 1.550 ± 0.184 | 0.22 |
| C22:6 n-3 | 6.092 ± 1.721 | 9.620 ± 3.473 | 8.443 ± 0.870 | 0.050 |

HERO, herring oil; ANCO, anchovy oil.

Data are presented as mean and standard deviation for *n* 6 rats in each experimental group. Groups are compared using one-way ANOVA followed by Tukey HSD post hoc test when appropriate. Means in a row with different letters are significantly different (*P* < 0.05). LOQ, level of quantification

**Supplemental table 8**: Fatty acids in brain, presented as g/100g fatty acids (mean values and standard deviations)

|  | Control group | HERO group | ANCO group | *P* ANOVA |
| --- | --- | --- | --- | --- |
| C14:0 | 0.135 ± 0.020 | 0.161 ± 0.019 | 0.148 ± 0.014 | 0.086 |
| C16:0 | 20.516 ± 2.453 | 21.312 ± 1.420 | 21.026 ± 2.438 | 0.83 |
| C17:0 | 0.185 ± 0.034 | 0.202 ± 0.037 | 0.186 ± 0.036 | 0.59 |
| C18:0 | 21.694 ± 1.547 | 21.717 ± 1.360 | 21.852 ± 1.539 | 0.98 |
| C20:0 | 0.752 ± 0.305 | 0.664 ± 0.171 | 0.687 ± 0.343 | 0.87 |
| C22:0 | 0.763 ± 0.423 | 0.620 ± 0.253 | 0.603 ± 0.369 | 0.72 |
| C23:0 | 0.744 ± 0.185 | 0.742 ± 0.176 | 0.731 ± 0.177 | 0.99 |
| C24:0 | 1.384 ± 0.941 | 1.077 ± 0.521 | 1.057 ± 0.718 | 0.72 |
| C16:1 n-7 | 0.343 ± 0.031^a^ | 0.420 ± 0.051^b^ | 0.394 ± 0.048^a^ | 0.042 |
| C16:1 n-9 | 0.130 ± 0.023 | 0.142 ± 0.025 | 0.139 ± 0.018 | 0.68 |
| C18:1 n-7 | 2.950 ± 0.909 | 2.784 ± 0.722 | 2.743 ± 0.778 | 0.91 |
| C18:1 n-9 | 15.219 ± 2.538 | 14.646 ± 1.545 | 14.803 ± 2.421 | 0.91 |
| C20:1 n-7 | 0.412 ± 0.312 | 0.278 ± 0.178 | 0.269 ± 0.312 | 0.83 |
| C20:1 n-9 | 1.749 ± 1.328 | 1.358 ± 0.768 | 1.502 ± 1.448 | 0.87 |
| C22:1 n-7 | 0.101 ± 0.105 | 0.073 ± 0.055 | 0.091 ± 0.105 | 0.87 |
| C22:1 n-9 | 0.185 ± 0.125 | 0.151 ± 0.082 | 0.175 ± 0.134 | 0.87 |
| C18:1 n-11 | <LOQ | <LOQ | <LOQ |  |
| C20:1 n-11 | <LOQ | <LOQ | <LOQ |  |
| C22:1 n-11 | <LOQ | <LOQ | <LOQ |  |
| C24:1 n-9 | 1.973 ± 1.531 | 1.458 ± 0.883 | 1.539 ± 1.541 | 0.80 |
| C18:2 n-6 | 1.661 ± 0.238 | 1.515 ± 0.258 | 1.523 ± 0.157 | 0.50 |
| C20:3 n-6 | 0.358 ± 0.036 | 0.423 ± 0.059 | 0.405 ± 0.023 | 0.084 |
| C20:4 n-6 | 10.168 ± 2.328 | 10.241 ± 1.978 | 10.48 ± 2.045 | 0.97 |
| C22:4 n-6 | 2.800 ± 0.576 | 2.600 ± 0.536 | 2.701 ± 0.409 | 0.81 |
| C22:5 n-6 | 0.385 ± 0.136 | 0.302 ± 0.095 | 0.312 ± 0.094 | 0.42 |
| C20:5 n-3 | 0.011 ± 0.004^a^ | 0.037 ± 0.012^b^ | 0.031 ± 0.022^b^ | 0.019 |
| C22:5 n-3 | 0.518 ± 0.020^a^ | 0.699 ± 0.025^b^ | 0.680 ± 0.020^b^ | 3.0x10^-9^ |
| C22:6 n-3 | 14.933 ± 1.921 | 16.340 ± 0.357 | 15.842 ± 2.072 | 0.38 |

HERO, herring oil; ANCO, anchovy oil.

Data are presented as mean and standard deviation for *n* 6 rats in each experimental group. Groups are compared using one-way ANOVA followed by Tukey HSD post hoc test when appropriate. Means in a row with different letters are significantly different (*P* < 0.05). LOQ, level of quantification
